# Psychometric evaluation of the Persian version of the Doloplus-2 (P-Doloplus-2) scale in elderly with dementia

**DOI:** 10.3906/sag-2001-117

**Published:** 2020-06-23

**Authors:** Mohammad ZARE, Zahra TAGHARROBI, Khadijeh SHARIFI, Zahra SOOKI, Javad ABOLHASANI

**Affiliations:** 1 Trauma Nursing Research Centre, Faculty of Nursing and Midwifery, Kashan University of Medical Sciences, Kashan Iran; 2 Department of Neurology, Faculty of Medicine, Kashan University of Medical Sciences, Kashan Iran

**Keywords:** Weights and measures, aged, dementia, pain, psychometrics

## Abstract

**Background/aim:**

A scale for behavioural pain assessment is useful for the detection and determination of pain in the elderly with dementia. This study aimed to translate and evaluate the psychometric properties of Doloplus-2 in the elderly with dementia in Iran.

**Materials and methods:**

In this methodological study, after translation and evaluating the face and content validity of Doloplus-2, 100 elderly people were selected by the convenience sampling method in Kashan, 2018–2019. Exploratory factor analysis, convergent validity, and known-groups comparison were applied to determine construct validity. Reliability was also assessed through internal consistency, equivalence, and stability methods were used. The ceiling and floor effects were also examined. Data were analyzed using the SPSS-v16 and via Mann-Whitney U test, Cronbach’s alpha, Spearman-Brown, and intraclass correlation coefficient (ICC).

**Results:**

The scale’s content validity index was 0.95%, and the item impact of each item was above 1.5. Factor analysis identified 2 “social-functional” and “conventional subjective-objective” factors in scale that altogether were able to explain 76% of the total variance. The results revealed that P-Doloplus-2 could discriminate between the 2 groups of elderly with and without known chronic painful diseases (P < 0.0001). There was a significant positive correlation between P-Doloplus-2 and PACSLAC-II-IR scores (r = 0.878, P < 0.0001). Cronbach’s alpha, ICC, and standard error of measurement for the scale were 0.950, 0.864, and ± 1.759, respectively. The frequency of minimum and maximum possible score of scale was less than 15%.

**Conclusion:**

The Persian version of Doloplus-2 can be considered as a valid and reliable scale for pain assessment in the elderly with dementia.

## 1. Introduction

Pain is a common problem among elderly people with dementia [1]. International studies have reported a pain prevalence of 17% to 79.5% in the elderly with dementia living in nursing homes [2]. Studies in Iran on community-dwelling elderly people reported a pain prevalence rate of 79.8%–82.4%, but no separate statistics have been reported for the elderly with dementia [3,4]. Pain occurs in the elderly for a variety of reasons, such as musculoskeletal disorders, ischemia, neuropathy, or malignancies [2]; if it continues, will cause severe consequences such as depression, anxiety, social isolation, sleep disorder, sedentary behaviour, reduced quality of life [5].

Despite the prevalence and significant consequences of pain in the elderly with dementia, pain in this group is often ignored and underestimated due to a variety of reasons, including addiction to analgesics, concern about the side effects of analgesics, and widely held misconceptions about the inevitability of pain among elderly people [6,7]. In addition, dementia can cause memory impairment, aphasia, apraxia, and agnosia and gradually affect all functional aspects of the individual. This issue can cause a greater tendency to ignore the pain in this group [6]. On the other hand, in some patients, pain appears in the form of some symptoms like aggression, restlessness, various mood disorders, hallucinations, and delusions that can sometimes be confused with dementia symptoms due to their similarities and may lead caregivers to use antipsychotic medications. This issue is of serious concern because of the possible side effects of taking such medicines [8]. Therefore, assessment and proper detection of pain and its severity are essential in implementing effective therapy in the elderly with dementia.

Regarding the subjective concept of pain, self-report is often represented as “the gold standard” in the assessment of pain [7,8]. Although this method of pain assessment is often used at the onset of dementia, its use will be questioned as the disease progresses and patients lose their ability to communicate. Therefore, considering the conditions of patients with dementia, behavioural symptoms of pain should be taken into account in the evaluation of pain in this group [8]. The American Geriatrics Society (AGS) has organized and presented behavioural pain indicators in 6 different behaviours in the elderly with dementia [9]. Based on which different tools were developed for pain assessment among these patients over the past few years [8,10]. In Iran, however, nonspecific pain rating scales such as a verbal descriptor scale (VDS) and faces pain scale (FPS) are used to measure pain in this group of patients [3,4]. Considering the incidence of aphasia, agnosia, and other sensory-motor disorders in patients with dementia, and the nature of these scales, their use in such patients are severely restricted [8]. Haghi et al. translated and evaluated the psychometric properties of the PACSLAC-II (the pain assessment checklist for seniors with limited ability to communicate-II) as a specific tool to measure pain in patients with dementia [11]; however, this tool has many items and some relatively specialized concepts, and this feature limits its use.

According to the findings of several studies conducted on pain assessment scales in the elderly with dementia, the Doloplus-2, a pain behavioural assessment scale, seems to be a useful scale in the diagnosis of pain and determination of pain severity. This scale can provide a wide range of behavioural pain indicators in dementia patients [9], can be easily used by professional caregivers [8], and its psychometric properties appear to be favourable in different communities [12–17].

Doloplus-2 was developed by Wary et al. in France in 2001 [12] to assess pain in nonverbal cognitively impaired older adults [18]. This scale developed for the multidimensional assessment of pain. It consists of 3 dimensions and a total of 10 items: somatic reactions (5 items), psychomotor reactions (2 items), and psychosocial reactions (3 items). Each item is levelled with 4 behavioural descriptions representing increasing severity of pain rated from 0 to 3. Individual item scores are summed to arrive at a total score ranging from 0 to 30 scores. Score 5 indicates pain [12,18]. Doloplus-2 as one of the most widely used pain assessment tools in patients with dementia have been currently translated into English, Dutch [13,19], Norwegian [14], Italian, Spanish, Portuguese [13], Japanese [15], and Chinese [16] and its psychoanalysis has been done. In all different translated versions, the desired level of psychometric properties has been verified.

By searching the national and international electronic scientific databases available to the authors of the article, no study has been conducted on translation, psychometric validation, or the use of the Doloplus-2 in Iran. Given the strengths of the scale, the present study was conducted to translate the Doloplus-2 into Persian and evaluate its psychometric properties, as a pain-behavioural assessment scale, among elderly people with dementia in Iran.

## 2. Materials and methods

This methodological study focusing on translation and psychometric evaluation of the Doloplus-2 scale was conducted in 2 phases in Kashan city, Iran, during 2018-2019.

### 2.1. Phase I: translation of the scale

At this phase, the Doloplus-2 scale was translated according to Wild et al. (2005) guideline [20]. Accordingly, in the first step (preparation), after gathering the required information about the intended scale, and getting permission from its developers by Email, translation and psychometric evaluation of the scale was performed. In the second step (forward translation), the English version of the Doloplus-2 was translated into Persian independently by 2 translators fluent in both Persian and English and familiar with health literature in the aging field. At the reconciliation step, the translated texts were reviewed in an expert panel consisting of members of the research team, and they reviewed the translations to achieve the best possible version. The Persian version of the scale approved by the expert panel, along with the original version, was given to a person with expertise in the related field and Persian literature to evaluate the scale in terms of appropriate equivalent vocabularies and grammar. Then, his points of view were reviewed by the research team, and the necessary corrections were made.

In the next step (back-translation), the primary form of the translated version was back-translated into English by a person fluent in English and Persian, familiar with relevant texts and independent of the individuals involved in the forward translation process. During the back-translation review, the Persian version, the original version, and the English back-translation were reviewed by the members of the research team, and disagreements were discussed, agreed upon by both the researchers and the back-translator and final revisions were made. At the harmonization step, the revised English translated version was sent to the designers of the scale to investigate the conceptual conformity of the revised version with the original version, and they approved the conformity of the new version. In the cognitive debriefing step, 10 caregivers (who were diverse regarding age, sex, education, and work experience) were recruited to assess whether the vocabularies used in the scale are appropriate and understandable. By conducting an individual and face-to-face interview with each of the caregivers, they were asked about each of the items, words that were not understandable, and their equivalents, which they believed to be more common and comprehensible. In the review of cognitive debriefing results and finalization, the research team made the necessary adjustments to the scale with respect to the questions and ambiguities raised by the caregivers in the cognitive stage. At the proofreading and final report step, a Persian version was finally reviewed by a research team and a Persian language editor in order to resolve the grammatical and writing problems, and a primary version of the Doloplus-2 scale was prepared to investigate its psychometric properties [20].

### 2.2. Phase II: psychometric evaluation of the scale

This phase was implemented in 4 steps:

#### 2.2.1. The first step: evaluation of face and content validity

For face and content validity assessments, the Persian version of the Doloplus-2 scale was provided to 10 experts in different fields of neurology, geriatrics, psychiatry, psychology, and psychometrics [21]. Regarding the qualitative assessment of the scale, they were asked to comment on the comprehensibility, grammaticality, literature, scoring, components, and totality of the scale, and adequacy, clarity, and simplicity of the items [22] and accordingly, the necessary changes were made.

Content validity was quantitatively assessed by calculating the content validity ratio (CVR), content validity index (CVI), and modified kappa statistic [21]. Experts were asked to rate the essentiality of each item on a 3-point Likert scale for CVR strict calculation and its relevance on a 4-point Likert scale for CVI and modified kappa statistic calculation [22]. The Lawshe table [23], Waltz and Bausell’s (1981) index [24], and Polit and Beck’s (2012) approach [25] were used to assessing the results of the CVR, CVI, and the modified kappa statistic, respectively. The S-CVI/Average was also used to calculate the total CVI [25].

For quantitative face validity assessment, the same experts were asked to rate the importance of each item on a 5-point Liker scale and then, their rating scores were used to calculate item impact score. Item impact score above 1.5 was considered to be optimal [21,26]. Regardless of the investigations carried out by the experts for qualitative assessment of face validity, each one of the 10 caregivers (who were diverse with respect to age, sex, education, and work experience) were asked to answer each question of the questionnaire separately and express their perception about each question. Then the respondents’ perceptions were compared with the main intention of the question [26].

#### 2.2.2. The second step: data collection to assess construct validity (factor analysis, known-groups comparison, and convergent validity), reliability and the ceiling and floor effects

Some statisticians believed that in factor analysis, the number of samples should be 5 to 10 times the number of tool items [27]; since the Doloplus-2 contains 10 items, the minimum number of samples needed was estimated to be 100, 10 samples for each item, in the sample size calculation formula. The inclusion criteria in this study were: having Iranian citizenship, age 60 and over, having dementia based on the neurologist diagnosis by interview and CT scan, lack of mental retardation based on the neurologist diagnosis, having hearing ability to cooperate in implementing a standard mobility protocol, satisfaction with participation in the study (by obtaining consent from the patient and his/her legal guardian or caregiver of the elderly), no use of analgesics 6 h before the assessment, permanent residency for at least 1 month for elderly people residing in nursing homes and having qualified caregivers (taking care of elderly with dementia at home for at least 4 days per week for at least 1 month or at least 4 shifts per week for at least 1 month in the elderly care centers). The exclusion criterion was the voluntary withdrawal of the participant or his/her caregiver/guardian from the study. Sampling was done by convenience sampling in 2 nursing homes and a private neurology clinic in Kashan from December 2018 to June 2019.

After obtaining the necessary authorizations from the relevant authorities, and receiving introduction letter from the vice chancellor for research and technology of the Kashan University of Medical Sciences, the researcher referred to two nursing homes and a private neurology clinic in Kashan, to extract a list of the elderly with dementia under the supervision of these centers and contact their legal guardians. If the elderly and his/her primary caregiver were eligible and consented to participate in the study, an appointment for assessment was made with each participant’s guardian. Then, we attended the appointment for data collection.

Data were collected by demographic questionnaire, the Persian-clinical dementia rating (P-CDR) [28], Doloplus-2 and PACSLAC-II-IR [11] through observation and interview with the patient and his/her caregiver and based on the patient’s record.

The demographic questionnaire included 8 questions, including age, sex, marital status, education, employment status, place of residence, history of known chronic painful diseases, and name of the diseases.

The Persian version of the CDR scale includes 75 questions, which should be asked from the participants and their companion in 6 areas, including memory, spatial or temporal orientation, judgment and problem solving, community affairs, home activities and leisure, and personal affairs. Each area scored on a scale from 0 to 3; the total score in the scale ranges from zero to 18. Scores of 0, 0.5–2, 2.5–4, 4.5–9, 9.5–15.5, and 16–18 are considered as normal cognitive status, suspected cognitive impairment, very mild cognitive impairment, mild cognitive impairment, moderate cognitive impairment, and severe cognitive impairment, respectively. Psychometric validation of ‎‎‎ the Persian version of this scale was carried out by Sadeghi et al. (1390) in Iran; the experts of the field confirmed its face and content validity, and its reliability coefficient using Cronbach’s alpha was estimated 0.73 [28].

The Doloplus-2 consists of a list of 10 items divided into 3 subgroups: 5 somatic reactions items (somatic complaints, protective body postures adopted at rest, protection of sore areas, expression, and sleep pattern), 2 psychomotor reactions items (washing and/or dressing, and mobility) and 3 psychosocial reactions items (communication, social life, and problems of behavioural). Each item is scored from 0 to 3, where 0 is “absent”, and 3 is “the highest score of the behaviour”. It gives a range from 0 to 30, with higher scores indicating more pain severity. The cut-off score between ‘pain’ and ‘no pain’ was set at 5, as recommended by the scale’s developers [12]. The concurrent validity of this scale has been verified by the VDS, pain assessment in advanced dementia (PAINAD), and the PACSLAC scales [8]. Cronbach’s alpha coefficient and intraclass correlation coefficient (ICC) in different studies were reported to be 0.67–0.87 and 0.75–0.97, respectively [12,13,29].

The original version of the PACSLAC-II scale was developed by Chan et al. (2014) to assess pain in elderly people with severe dementia [30] and translated into the Persian language by Haghi et al. (2019) [11]. The Persian version of this scale contains 30 items, each item being rated as 0 (no pain) or 1 (pain). This scale scores the pain from 0 to 30, with a higher score indicating more severe pain behaviour. Its validity has been verified by factor analysis and concurrent validity. Also, the ICC between the raters was estimated to be 0.76 [11]. In the present study, the reliability of this scale by computing the Kuder-Richardson 21 coefficient (KR21) was 0.78.

The P-CDR was used to determine the severity of dementia in all the samples. The participants were examined at rest and during the standard mobility protocol presented by Husebo et al. using the pain assessment scales (Doloplus-2 and PACSLAC-II-IR) (Table 1) [31]. If the patient was not cognitively or physically able to perform the steps of the protocol, a qualified caregiver was recruited to help him/her. If either of them refused each of the steps, the participant was excluded from the study.

**Table 1:** Standard mobility protocol of Husebo et al. (2010).

Step	Actions
1	To open both hands (one hand at a time)
2	To stretch both arms towards the head (one arm at a time)
3	To stretch and bend both knees and hips (one leg at a time)
4	To turn in bed to both sides
5	To sit at the bedside

In order to increase accuracy in completing the pain assessment scale at rest and during the mobility protocol, participants were videotaped.

#### 2.2.3. The third step: evaluation of construct validity (factor analysis, known-groups comparison, and convergent validity) and the ceiling and floor effects

After data collection, construct validity was evaluated by exploratory factor analysis, known-groups validity, and convergent validity. Principal axis factoring (PAF) method with varimax rotation was used to extract data in exploratory factor analysis. Eigenvalue above 1 and the scree plot were used to determine the number of factors. The minimum load factor was estimated to be 0.52 based on Formula 1 in which “n” is the sample size [22]. Regarding the common factor loadings, the bigger factor loading was considered.

Critical Value = 5.152 ÷ √(n - 2)

Formula 1

To compare the known-groups, the elderly with dementia were divided into 2 groups with known chronic painful diseases and the nondiseased. Then, the scores obtained for the Doloplus-2 were compared between the 2 groups. The Persian version of the Doloplus-2 and the PACSLAC-II-IR scale were completed simultaneously for all participants in the convergent validity assessment.

The ceiling and floor effects of the scale were assessed [32]; so that the ceiling and floor effects were evaluated based on the relative frequency of the samples with the highest and lowest possible attainable scores, respectively.

#### 2.2.4. The fourth step: reliability assessment

The reliability of the Persian MPS was assessed via the internal consistency, equivalence, and stability. 

The internal consistency of the scale was evaluated by calculating Cronbach’s alpha coefficient.

For equivalence assessment, the scale was completed for 20 samples selected from the elderly of nursing homes simultaneously and independently by 2 individuals (first author of the paper and one trained caregiver), and the interrater agreement was estimated.

The standard error of measurement (SEM) was also calculated to evaluate the stability of the scale [33].

### 2.3. Data analysis 

Data were analyzed using the SPSS software version 16. Quantitative variables were described by using central tendency and dispersion indices and categorical variables described by absolute and relative frequencies. Additionally, CVI, CVR, and modified kappa statistic were used for evaluating quantitative content validity, and quantitative face validity was analyzed using an impact score method. The Kolmogorov-Smirnov test analyzed the normality of quantitative data. The Kaiser-Meyer-Olkin (KMO) and Bartlett’s test of sphericity were used to determine the suitability of the data for factor analysis. Spearman-Brown test was used to investigate the correlation between the scores of the Persian version of Doloplus-2 with the PACSLAC-II-IR scale, Mann-Whitney U test for comparing known-groups, Cronbach’s alpha coefficient for assessing internal consistency, and ICC and the weighted kappa were used to examine the ICC.

The SEM was calculated based on Formula 2, where “SD” is the standard deviation of the scores, and “r” is the Cronbach’s alpha coefficient [33]. In all the analyses, the significance level was set at P < 0.05.

SEM = SD √1 - r

Formula 2

## 3. Results

### 3.1. The translation phase of the scale

The draft of the Persian version of the Doloplus-2 had 10 items with a 4-point Likert scoring style (Appendix 1).

### 3.2. Psychometric evaluation of the scale

#### 3.2.1. Evaluation of face and content validity

In qualitative content validity assessment, minor modifications were made to the draft of the Persian version of the Doloplus-2; for example, in one of the items, the phrase of “Reduce his/her walking distance” was changed to the phrase of “Has lower mobility”. In quantitative content validity assessment, the CVR of all the scale items ranged from 0.8 to 1, which is higher than the number in the Lawshe table (0.62 for 10 experts). The CVI calculated for each item ranged from 0.8 to 1, which is higher than the minimum acceptable value based on the Waltz and Basel index (0.79). The modified kappa statistic also ranged from 0.791 to 1, which was higher than 0.74. The S-CVI/Average was estimated to be 0.95. In the qualitative face validity phase, no changes were made on the scale. In the quantitative face validity assessment, the impact score of all items was above 1.5 (3.44 to 5).

#### 3.2.2. Evaluation of construct validity (factor analysis, known-groups comparison, and convergent validity) and the ceiling and floor effect

A total of 127 older adults were included in the current study. Of whom, 24 cases declared (by yourself or by their guardians) that they were not willing to participate in the study, and 2 persons were not included in the study because of concomitant mental retardation; 1 person was excluded from the study due to dissatisfaction of his/her legal guardian. Finally, data of 100 samples were analyzed. The age range of the participants was from 65 to 93 years, with a mean age of 87.310 (± 8.497) years. Fifty-four percent of the participants were residing in their homes, and 27% had mild dementia (Table 2).

**Table 2:** Demographic characteristics of samples (n = 100).

Characteristics		n	%	Characteristics		n	%
Sex	Male	29	29	Place ofresidence	Home	54	54
	Female	71	71		Nursing home	46	46
Marital status	Single	14	14	EmploymentStatus	Employed	0	0
	Married	34	34		Unemployed	3	3
	Divorced/separated	12	12		Retired	11	11
	Widowed	40	40		Disabled	86	86
Education	Illiterate	76	76	Severity ofDementia	Mild	27	27
	Read and write	6	6		Moderate	53	53
	Elementary school	8	8		Severe	20	20
	High school	5	5	Known chronic painful diseases	Yes	37	37
	Above diploma	5	5		No	63	63
Type of known chronic painful diseases* (n = 37)	Skin and mucosal ulcers (such as pressure ulcers and skin cuts)					26	70.27
	Musculoskeletal disorders (such as arthritis, vertebral disc, spinal stenosis, fibromyalgia, muscle tension, etc.)					12	32.43
	Rheumatic diseases (such as rheumatoid arthritis, osteoarthritis, gout, lupus, etc.)					7	18.92

* The relative prevalence of the types of known chronic painful diseases was calculated in 37 samples of these diseases. Since some people had more than 1 known chronic painful diseases, the sum of the percentages was more than 100.

Regarding the suitability of the scale for performing factor analysis, KMO indicated the adequacy of the number of samples (0.870); the Bartlett test also showed that the interitem correlation matrix had no problem for analysis (χ2 = 1125.943, P < 0.0001). Factor analysis resulted in the extraction of 2 factors. The “functional-social” dimension (7 items) with an Eigenvalue of 4.386 and the “conventional subjective-objective” dimension (3 items) with an Eigenvalue of 3.228 were able to explain 43.859% and 32.283% of the total variance, respectively. These factors altogether could explain 76.142% of the total variance (Table 3 and Figure).

**Table 3:** Communalities and factor loadings of items of the extracted factors in the Persian version of the Doloplus-2.

No.	Item theme	Extracted factors*		Communalities
		First**	Second***	
1	Somatic complaints		0.908	0.912
2	Protective body postures adopted at rest	0.733		0.764
3	Protection of sore areas	0.611		0.546
4	Expression		0.909	0.908
5	Sleep pattern	0.762		0.655
6	Washing and/or dressing	0.824		0.695
7	Mobility	0.788		0.862
8	Communication	0.728****	0.580	0.867
9	Social life	0.794		0.816
10	Problems of behavioural		0.571	0.592

* The minimum factor loaded for each item was set 0.52. Factor loadings less than 0.52 were not inserted in the table. ** Considering the content of the items, the first factor (including questions 2, 3, 5, 6, 7, 8, and 9) was named the “social-functional” dimension. *** Regarding the content of the items, the second factor (including questions 1, 4, and 10) was named the “conventional subjective-objective” dimension. **** In relation to the common loading factor, the item was loaded onto a factor having a larger loading factor.

**Figure:**
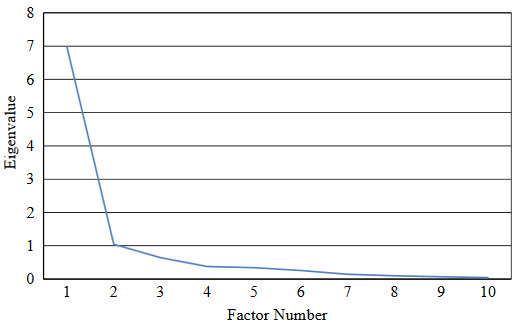
Scree plot of the Persian version of the Doloplus-2 scale.

In the known-groups comparison analysis, the pain intensity determined by the Persian version of the Doloplus-2 in the 2 groups with and without known chronic painful diseases were 18.270 (± 5.738) and 6.920 (± 5.589), respectively, which the results of the Mann-Whitney U test showed a significant difference (P < 0.0001).

In the convergent validity analysis, the scores obtained from the Doloplus-2 and PACSLAC-II-IR scales for the studied items were 11.120 (7.865) and 11.260 (5.425), respectively. There was a significant and positive correlation between these 2 scores (r = 0.878, P < 0.0001). Also, there was a significant positive correlation between the first and second factors of the Persian version of the Doloplus-2 with the PACSLAC-II-IR scale (r = 0.832 and r = 0.808, respectively, P < 0.0001).

As with the results of the floor and ceiling effects analyses, the relative frequencies of the minimum and maximum possible scores obtained from the Persian version of the Doloplus-2 were 2% and 0% (less than 15%), respectively.

#### 3.2.3. Evaluation of reliability

In the internal consistency analysis, the results showed that a Cronbach’s alpha coefficient for the total scale was 0.950 and for the first and second factors were 0.944 and 0.903, respectively.

In the equivalence analysis for the total score of scale, the ICC coefficient between the scores of the raters was 0.864 (CI95%: 0.655–0.946, P < 0.0001). Also, for each item, the coefficient of agreement between the raters (weighted kappa) ranged from 0.640 to 0.919 (P < 0.0001) (Table 4).

**Table 4:** The weighted kappa coefficient of the items in the Persian version of the Doloplus-2 (2 raters and 20 samples).

No.	Item theme	Kappa*	SE	Confidence interval 95%		P-value
				Lower bound	Upper bound	
1	Somatic complaints	0.643	0.167	0.315	0.971	0.003
2	Protective body postures adopted at rest	0.640	0.164	0.319	0.961	0.003
3	Protection of sore areas	0.687	0.104	0.482	0.891	0.002
4	Expression	0.759	0.068	0.626	0.892	0.001
5	Sleep pattern	0.750	0.080	0.594	0.906	0.001
6	Washing and/or dressing	0.856	0.066	0.727	0.985	< 0.0001
7	Mobility	0.824	0.094	0.639	1.000	< 0.0001
8	Communication	0.919	0.053	0.815	1.000	< 0.0001
9	Social life	0.866	0.082	0.706	1.000	< 0.0001
10	Problems of behavioural	0.848	0.060	0.731	0.966	< 0.0001

* The weighted kappa coefficient calculated for each item is of quadratic type.

The SEM of the scale was ± 1.759.

## 4. Discussion

The present study aimed to translate and assess the psychometric properties of the Doloplus-2, as a behavioural pain assessment scale, in the elderly with dementia in Iran. It was found that the Persian version of the scale could determine pain severity in a range of 0 to 30 and had a desirable validity and reliability in the target population.

In the translation phase of the scale, an attempt was made to maintain the maximum semantic and technical similarity of the text using the optimal translation equivalents and standard translation. If there are semantic equations, cognitive and perceptual terms in the translation process, it can be argued that this tool is consistent with the principles of cultural adaptation and is intended for the target group to understand the phrases correctly and easily [34].

The results of qualitative content validity showed that the items were approved by the relevant experts in terms of intelligibility, grammar, literature, clarity, and simplicity. Also, considering the desirable values obtained from the calculation of the CVR (above 0.62), CVI (above 0.79) and modified kappa statistic (above 0.74), it can be claimed that the criteria for content validity are met in the Persian version of the scale [21].

In qualitative face validity analysis, the scale items were not changed, which confirms the appropriateness of the items of the Persian version of the scale from the viewpoint of the experts and caregivers [34]. Unchanged items at this stage may be due to the use of Wild et al. (2005) guideline for the translation of the scale since the cognitive debriefing of the scale is one of the stages of its completion [20]. Also, quantitative face validity assessment indicated that the item impact scores of all items were more than 1.5, which confirms the face validity of the scale. Therefore, the face validity of the Persian version of the Doloplus-2 is confirmed.

Construct validity through exploratory factor analysis identified 2 factors in the Persian version of the Doloplus-2, namely “social-functional” and “conventional subjective-objective”. The social-functional dimension includes items that examine the individual and social functioning of older adults with dementia, such as sleep, mobility, and communication patterns; Whereas the conventional subjective-objective dimension refers to the most common symptoms of pain, such as physical complaints and altered facial expression. The extracted factors were more than 43% and 32%, respectively, and could explain over 76% of the total variance. These numbers, according to Wipulanusat et al. (2017), indicated excellent construct validity [35]. Therefore, the construct validity of the Persian version of the Dololplus-2 scale was confirmed. The results of the study by Neville and Ostini (2014) showed that Doloplus-2 is a single-factor scale. Also, the percentage of variance explained in their study was lower than in the present study [29]; however, unlike the present study, the data were collected by multiple raters. In a study aimed to assess the psychometric properties of the Chinese version of Doloplus-2, 3 factors were identified in the scale that was able to explain 65% of the variance of the score [16]. Regardless of the differences in the severity of dementia in the studied samples, the use of multiple raters for pain assessment in the studies can be the cause of this difference.

The results of the construct validity assessment using known-groups comparison showed that the Persian version of Doloplus-2 could discriminate between the 2 groups, with and without known chronic painful diseases. Therefore, it can be noted that the scale is able to detect the pain and its severity.

 Convergent validity showed a strong, positive, and significant correlation between total score and the score of each subscale of the Persian version of the Doloplus-2 with the PACSLAC-II-IR scale. Many researchers believe that a correlation of 0.5 indicates strong convergent validity [22]. Some experts, however, do not consider those too high coefficients to be desirable and accordingly emphasize that both scales (the existing and new scales) are the same, and there is no need to introduce new ones. However, they acknowledge that the new scale due to its nature can be a good alternative scale [36]. Although the Persian version of the Doloplus-2 (with 10 items) has fewer items than the PACSLAC-II-IR scale (with 30 items), it covers a wide range of behavioural pain indicators; therefore, its use for accurate assessment of pain by caregivers and researchers seems to be preferable. The results of Pautex et al. (2007) study confirmed the convergent validity of the Doloplus-2 and visual analogue scale (VAS), which are in line with the results of the present study [17].

The relative frequency of the minimum and maximum scores obtained from the Persian version of the Doloplus-2 were less than 15%, meaning that the scale had no ceiling and floor effects [32]. The lack of ceiling and floor effects refer to including items on the scale that represent the maximum and minimum intensity of the pain, respectively. The existence of appropriate items can confirm the content validity and stability of the scale [22,32].

Cronbach’s alpha coefficient was 0.95 for the total scale and above 0.9 for each of its subscales. Given that the coefficient is higher than 0.7 [37], it can be stated that the scale has good internal consistency. Internal consistency of the Doloplus-2 has been reported in studies ranging from 0.58 to 0.87 [8,12,16,19,29]. The reason for the difference between the results of these studies and the present study may be because of differences in the number and characteristics of the studied samples, including age, severity of dementia, residence of place, and other demographic information.

The ICC of the Persian version of the Doloplus-2 was 0.864. Also, the calculated kappa coefficient for each item was between 0.640 and 0.919. Koo and Li (2016) consider the ICC of 0.6 or more as acceptable reliability [38]. Therefore, the results show that the coefficient of agreement between the raters is desirable. The coefficient of agreement between the raters was reported between 0.47 and 0.96 in similar studies [8,13,15,16,29]. The difference between the results of these 2 studies may be due to the quality of the training provided to the raters (on how to complete the scale). Because of the nature of the concepts and terms in the Doloplus-2 scale, in order to achieve the desired results, it is necessary to educate caregivers on how to complete the scale which is focused on in this study. With respect to each item, the kappa coefficients of the items were reported from 0.19 to 1.00 in similar studies [13,29]. Regardless of the number of raters and the quality of training provided to raters on how to complete the scale, the method of calculating the reported kappa coefficient should also be taken into account when comparing the findings of different studies.

The assessment of the absolute reliability of the Persian MPS revealed a standard error of measurement of ± 1.795, which indicates that if the scale is completed again for an individual, its score may be changed by ± 1.795. The scoring range of the Persian version of the Doloplus-2 was from 0 to 30, which is low, and thus supports the stability of the scale [22].

Variation in the characteristics of the units under the study is one of the strengths of the current study. Regardless of the relatively small sample size, considering the known cases of the chronic painful disease can be considered as a limitation of this study. In future studies, it is recommended that confirmatory factor analysis be performed on the Persian version of the Doloplus-2. Also, it is recommended that a study be repeated with larger sample size and with respect to the severity of dementia.

The P-Doloplus-2 scale has 10 items and can be used as a valid and reliable scale for assessment of pain in patients with dementia by caregivers and researchers; it can be used after receiving adequate training on how to complete the scale.

## Acknowledgement/Disclaimers

The authors would like to express their gratitude to the developers of the original version of the scale, all the elderly participating in the study, their caregivers and families, as well as the vice-chancellor of Research and Technology of Kashan University of Medical Sciences.

This research is a part of the first author’s thesis in the Master’s degree in Geriatric Nursing, which has been sponsored by the vice-chancellor of Research and Technology of Kashan University of Medical Sciences (Registration No: 9728).

## Conflicts of interest

The authors have no conflict of interest.

## Informed consent

The written permission was obtained from Vice Chancellor for Research and Technology (Registration No: 9728) and the Ethics Committee of Kashan University of Medical Sciences (No: IR.KAUMS.NUHEPM.REC.1397.015). In addition to obtaining written informed consent from the legal guardian of the elderly with dementia (if available) or head of the center responsible for the care of the elderly (if you do not have access to their legal guardian), informed consent was directly obtained from the elderly by taking into account the elderly’s conditions. The reasons for recording the videotapes and the confidentiality of them and other patients’ information were explained to the elderly’s caregivers and their legal guardians and finally their consent was obtained. If the legal guardian did not have consent to take out the videotapes from the elderly residents, the videotapes would be reviewed at the elderly residence, and the relevant files would be deleted after completing the scale. All the elderly people or their guardians had the right to change your mind and leave the research at any time.
